# COVID-19: a brief update for radiologists

**DOI:** 10.1590/0100-3984.2020.0074

**Published:** 2020

**Authors:** Gustavo de Souza Portes Meirelles

**Affiliations:** 1 Grupo Fleury, São Paulo, SP, Brazil.

**Keywords:** Coronavirus infection/diagnostic imaging, Coronavirus, Review, Infecção por coronavírus/diagnóstico por imagem, Coronavírus, Revisão

## Abstract

Coronavirus disease 2019 (COVID-19), which is caused by a new coronavirus-severe acute respiratory syndrome coronavirus 2 (SARS-CoV-2)-is a pandemic with major impacts on the health care sector, and a broad view of the disease is of fundamental importance for any radiologist. The purpose of this review is to address the main clinical and imaging aspects of COVID-19, as well as guidelines for requesting and using imaging methods; measures to protect patients and health care professionals; systems for quantifying pulmonary findings and preparing integrated reports; and the main innovations that have emerged during this pandemic.

## Abstract (pt)

A COVID-19, doença causada por um novo coronavírus (SARS-CoV-2), é uma pandemia que tem causado grandes impactos no setor de saúde, sendo fundamental para o médico radiologista uma visão ampla da doença. O objetivo desta revisão é abordar os principais aspectos clínicos e imaginológicos da COVID-19, assim como as diretrizes para solicitação e utilização dos métodos de imagem, medidas de proteção a pacientes e profissionais de saúde, sistemas de quantificação dos achados pulmonares e de elaboração de relatórios integrados e as principais inovações que têm surgido neste momento de pandemia.

## INTRODUCTION

In December 2019, in the city of Wuhan, China, there were reports of cases of pulmonary infection with a new coronavirus, designated severe acute respiratory syndrome coronavirus 2 (SARS-CoV-2). The disease, now known as coronavirus disease 2019 (COVID-19), soon spread to other cities in China, to other countries in Asia, and, subsequently, to all continents, prompting the World Health Organization to declare it a pandemic on March 11, 2020^(1)^. At this writing, there have been more than 12,500,000 confirmed cases of and 560,000 deaths from COVID-19 worldwide, Brazil having accounted for more than 1,800,000 cases and 70,000 deaths^(2)^.

The purpose of this review was to address the main clinical and imaging aspects of COVID-19. In addition, we discuss guidelines for requesting imaging methods; examine measures to protect patients and health professionals; detail systems for quantifying pulmonary findings and preparing integrated reports; and describe the main innovations that have emerged during this pandemic.

## CLINICAL ASPECTS

The clinical presentation of COVID-19 can be nonspecific, with symptoms common to other flu-like syndromes^(3)^. The majority (approximately 80%) of COVID-19 cases are mild, with limited symptoms and without evidence of viral pneumonia or hypoxia. In 15% of cases, patients evolve to moderate forms of the disease, with clinical signs of pneumonia (fever, cough, dyspnea, and tachypnea), although without signs of severe pneumonia (peripheral oxygen saturation > 90% on room air). Only 5% of patients develop the severe forms of the disease, showing not only clinical signs of pneumonia (fever, cough, dyspnea, and tachypnea) but also at least one of the following: peripheral oxygen saturation < 90% on room air; respiratory rate > 30 breaths/min; or severe respiratory disorder. A small proportion of patients with COVID-19 become critically ill, progressing to respiratory failure, cardiovascular shock, acute kidney injury, or acute liver failure. Individuals who are at a higher risk of developing the severe forms of the disease include those over 60 years of age and those who have comorbidities, such as diabetes mellitus, arterial hypertension, and cardiovascular disease^(4,5)^.

The main symptoms of COVID-19 are fever (occurring in 83-99% of patients), cough (in 59-82%), fatigue (in 44-70%), anorexia (in 40-84%), dyspnea (in 31-40%), myalgia (in 11-35%), as well as sore throat, nausea, dizziness, diarrhea, headache, vomiting, and abdominal pain; another symptom is anosmia, which has been reported by up to two thirds of patients and may be the only symptom in children and young adults^(5)^. These symptoms are similar to those of other viral respiratory diseases, although findings such as myalgia, sore throat, nausea, vomiting and diarrhea may suggest infection with a virus other than SARS-CoV-2^(6)^.

The average incubation period of infection with SARS-CoV-2 is 4-5 days, the maximum being 14 days. Most patients (97.5%) present at least one symptom within 11.5 days after infection. The test considered the gold standard for the diagnosis of infection with SARS-CoV-2 is reverse-transcriptase polymerase chain reaction (RT-PCR), which consists of direct detection of viral RNA in a respiratory sample collected from the nasopharynx, oropharynx, or lungs. Although the test has very high specificity, its sensitivity is low (60-70%), especially in the first three days after infection^(7)^.

## GUIDELINES FOR REQUESTING IMAGING EXAMINATIONS

Most medical societies do not recommend the use of imaging methods for screening patients under clinical suspicion of COVID-19. The American College of Radiology recommends the use of chest computed tomography (CT) only for symptomatic inpatients and that of portable chest X-ray in specific cases, such as inpatients who need imaging follow-up^(8)^. The recommendations emphasize the fact that a normal chest CT examination does not exclude a diagnosis of COVID-19 and that an altered examination does not confirm the clinical suspicion of the disease.

In a joint position statement, the Society of Thoracic Radiology and the American Society of Emergency Radiology, also advised against the routine use of chest CT to screen patients with suspected COVID-19^(9)^. Both societies recommend that chest CT be used only for patients with COVID-19 confirmed by laboratory tests and with suspected complications such as lung abscess and pleural empyema.

The Fleischner Society, which is composed of radiologists, pulmonologists, pathologists, and surgeons from different countries, also does not recommend chest CT for asymptomatic patients with COVID-19 or for those with only mild symptoms, except when there is suspicion of disease progression^(10)^. The recommendation of the society is that chest CT be performed in patients with moderate or severe COVID-19, in those with worsening respiratory status, and in those who present with hypoxemia or functional loss after having recovered from the disease.

The Colégio Brasileiro de Radiologia e Diagnóstico por Imagem (CBR, Brazilian College of Radiology and Diagnostic Imaging), in the latest version of its recommendations for the use of imaging methods in patients suspected of having COVID-19^(11)^, does not recommend the isolated use of chest CT for the screening or diagnosis of the disease. According to the CBR, the diagnosis of COVID-19 must be based on clinical and epidemiological data, together with the results of RT-PCR, serology tests, or both. Chest CT, always in correlation with clinical and biochemical data, can assist in defining the diagnosis. The CBR guidelines do not recommend the use of imaging examinations in asymptomatic patients. For mildly symptomatic patients with negative results on RT-PCR or no anti-IgM antibodies on serology, the guidelines recommend no imaging examination, although chest CT can be performed if the respiratory symptoms worsen. For mildly symptomatic patients who test positive for infection on RT-PCR or for anti-IgM antibodies on serology, the guidelines recommend that the risk factors for disease progression be evaluated: if none of those risk factors are present, no imaging examination is indicated unless the respiratory symptoms worsen; if any of the risk factors are present, chest CT can be performed. For mildly symptomatic patients who do not have access to laboratory tests, the CBR guidelines recommend estimating the pretest probability of COVID-19. If the pretest probability is low, no imaging method is indicated. If the pretest probability is moderate or high, the risk factors for disease progression should be assessed: if none of those risk factors are present, no imaging examination is indicated unless the respiratory symptoms worsen; if any of the risk factors are present, chest CT can be performed. Finally, for patients with moderate or severe symptoms, imaging examinations can be requested. According to the CBR guidelines^(11)^, imaging can also be performed when there is a need to assess complications such as pulmonary thromboembolism and bacterial infection.

For incidental findings of changes suggestive of COVID-19 in extrathoracic imaging examinations, such as CT or magnetic resonance imaging (MRI) of the spine, neck, or abdomen or oncologic positron emission tomography/CT (PET/CT), the radiologist should evaluate the findings and inform the attending physician of suspected changes consistent with COVID-19. Patients should be informed of the findings and instructed to contact their attending physician. As soon as changes suggestive of COVID-19 are detected on extrathoracic imaging examinations, personal protective equipment (PPE) must be provided for the patients and for the health professionals who are to proceed with the care of those patients^(12,13)^.

## PROTECTION OF PATIENTS AND HEALTH PROFESSIONALS DURING IMAGING EXAMINATIONS

During the current (COVID-19) pandemic, it is essential that adequate protection be provided for the affected patients and for the health professionals involved in their care. Patients with respiratory symptoms should receive a surgical mask to protect other contacts and health care teams, be instructed to clean their hands with alcohol-based hand sanitizer, and be referred directly to the sector where the imaging examination is performed, priority being given to their care. Health professionals who will provide patient care in the imaging sector (technical, medical, and nursing teams) should seek to maintain a safe distance from the patient and should wear full PPE, consisting of a surgical mask (N95 mask for aerosol-generating procedures), disposable long-sleeved gown, cap, goggles and disposable gloves. At the end of the imaging examination, terminal cleansing of the examination room and equipment must be carried out, and the cleaning crew must use full PPE as described above; if aerosol-generating procedures have been performed or the patient has removed the mask, in addition to the terminal cleansing of the room, it is recommended that the room be left unoccupied for at least 30 min before the next examination^(14,15)^.

## IMAGING ASPECTS OF COVID-19

### Chest X-ray

Chest X-ray is the simplest, most practical, and least expensive test to perform in patients with suspected COVID-19. In addition, the portability of the method makes it quite useful in bedridden patients and in specific situations, such as field hospitals, and can be used in order to monitor disease progression, to assess tracheal tubes/drug infusion lines, and to rule out complications such as pneumothorax, pneumomediastinum, and subcutaneous emphysema^(16)^.

**Figure 1:**
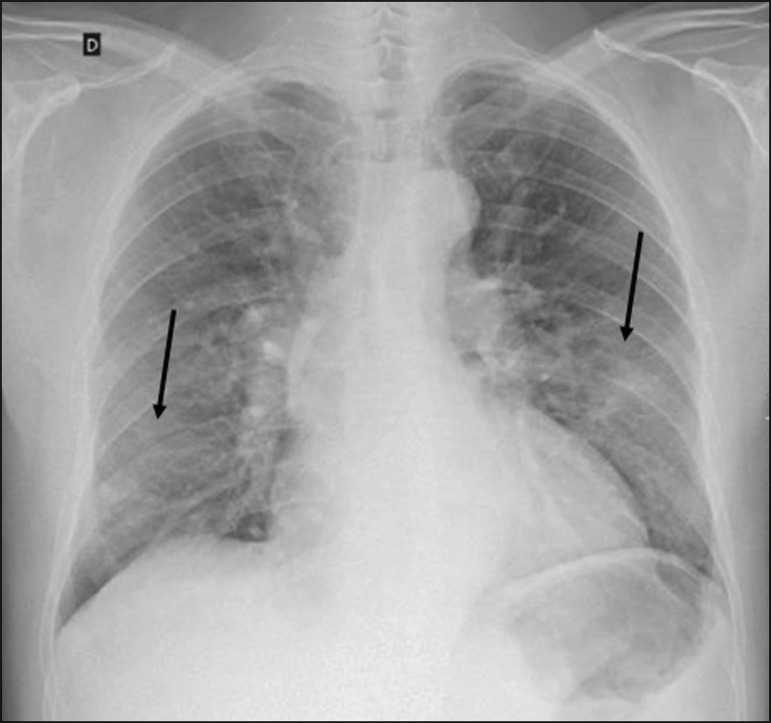
Chest X-ray of a 69-year-old male patient with a 2-day history of cough, adynamia, and fever who tested positive for SARS-CoV-2 on RT-PCR, showing low-attenuation pulmonary opacities (arrows) in the lung bases, more pronounced on the left.

Despite its availability and ease of execution, chest X-ray has low (30-69%) sensitivity in the evaluation of patients under clinical suspicion of COVID-19, and the findings are often normal in patients with mild forms of the disease. On chest X-rays that show alterations, the main findings (Figure 1) are consolidations (in 36-47% of patients) and low-density opacities (in 20-33%), typically with a peripheral distribution in the lung bases. Other findings, such as pleural effusion, are uncommon. In a study conducted by Wong et al.^(16)^, pleural effusion was observed in only 3% of patients. The findings peak 10-12 days after the onset of symptoms, and the pulmonary changes can progress rapidly, advancing to the middle and upper lung fields or evolving to diffuse pulmonary impairment similar to the diffuse alveolar damage seen in acute respiratory distress syndrome^(16,17)^.

### Chest CT

In patients with suspected COVID-19 for whom an imaging study is necessary, chest CT is considered the method of choice. According to a meta-analysis of 63 articles, carried out by Kim et al.^(18)^, chest CT has a sensitivity of 94%, a specificity of 37%, a positive predictive value of 1.5-30.7%, and a negative predictive value of 95.4-99.8%. Therefore, in regions where the prevalence of the disease is low, the use of chest CT is associated with a high number of false positives, which can increase medical costs, lead to additional tests or unnecessary treatments, and generate greater anxiety for patients. In regions where the prevalence of the disease is high, the use of chest CT should follow the guidelines of medical specialty societies^(18)^. A chest CT examination is usually performed without the use of intravenous contrast, except when complications such as pulmonary thromboembolism are suspected, in which case it is necessary to acquire contrast-enhanced images^(19,20)^.

On chest CT examinations of patients with COVID-19, the main findings are ground-glass opacities, crazy-paving pattern, consolidations, reticular opacities, subpleural lines, inverted halo sign, and pleural thickening (in 32% of cases). Less common findings include changes in the airways, vascular dilation, pulmonary nodules, lymph node enlargement (in 4-8% of cases), pleural effusion (in 5-15%), and pericardial effusion (in 5%), the latter three usually indicating a worse prognosis, either due to decompensation of pre-existing heart disease or the development of acute heart failure, arrhythmias, or acute cardiovascular injury^(19-22)^.

**Figure 2:**
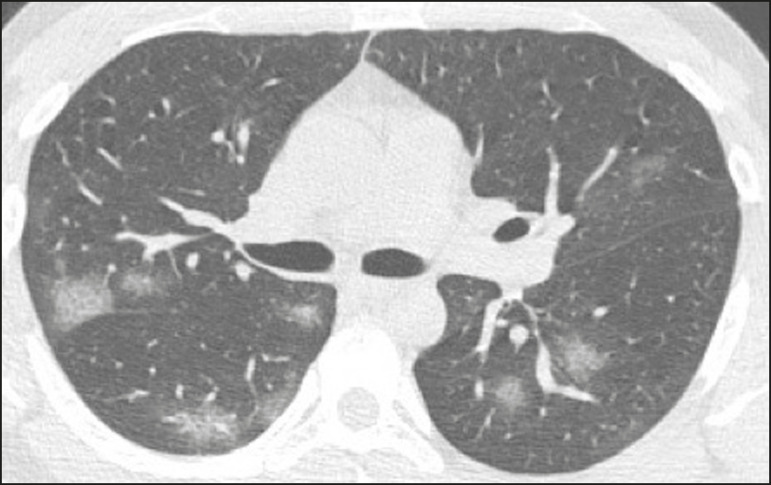
Chest CT scan of a 34-year-old female patient with a 4-day history of chest pain and cough who tested positive for SARS-CoV-2 on RT-PCR, showing bilateral ground-glass opacities with a predominantly peripheral distribution.

**Figure 3:**
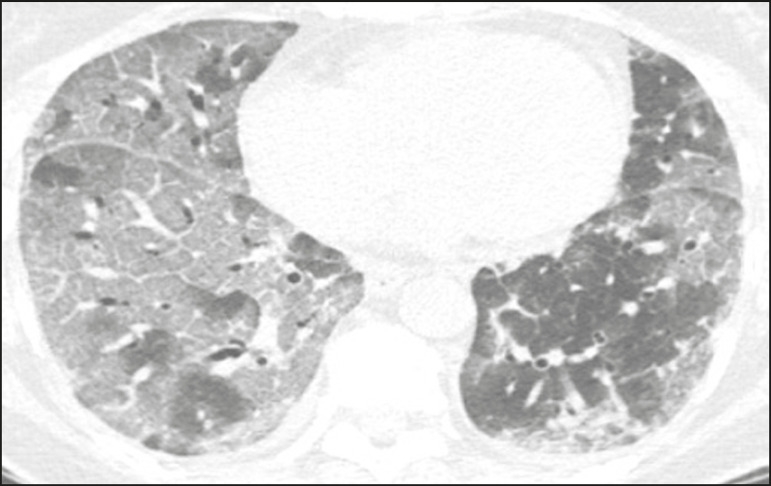
Chest CT scan of a 52-year-old male patient with a 6-day history of anosmia and dry cough who tested positive for SARS-CoV-2 on RT-PCR, showing a bilateral crazy-paving pattern, with ground-glass opacities and interlobular septal thickening.

**Figure 4:**
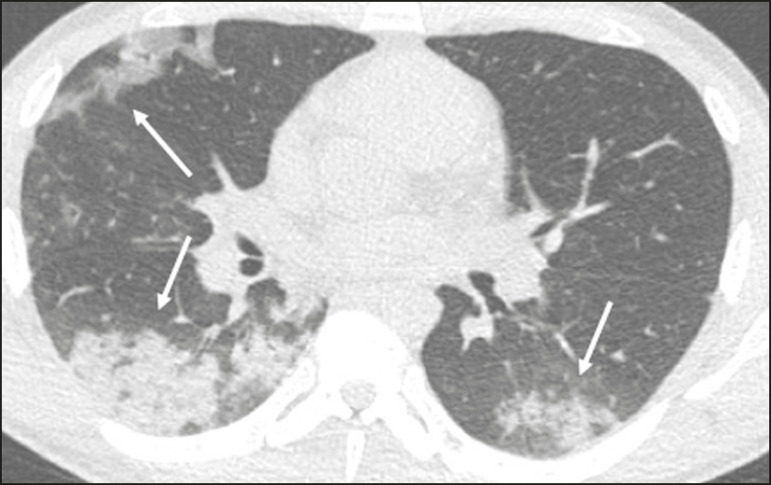
Chest CT scan of a 63-year-old male patient with a 7-day history of dyspnea and episodes of fever who tested positive for SARS-CoV-2 on RTPCR, showing bilateral peripheral pulmonary consolidations (arrows).

**Figure 5:**
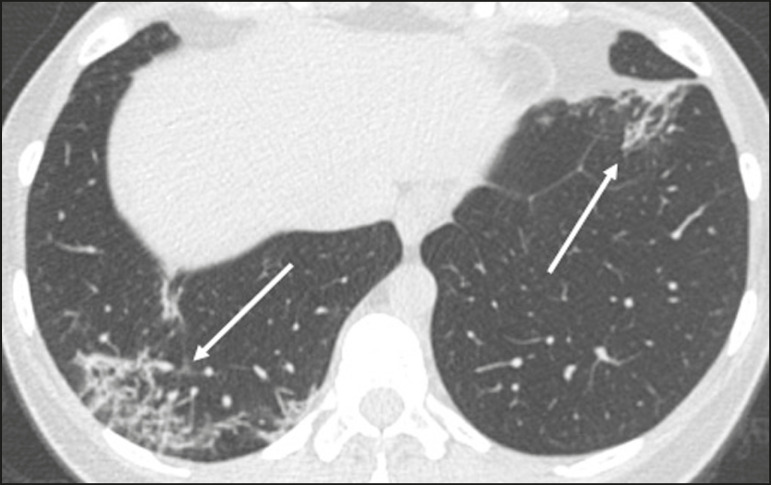
Chest CT scan of a 71-year-old female patient with an 8-day history of dyspnea and dry cough who tested positive for SARS-CoV-2 on RT-PCR, showing bilateral reticular opacities (arrows), with a peripheral distribution, in the lung bases.

**Figure 6:**
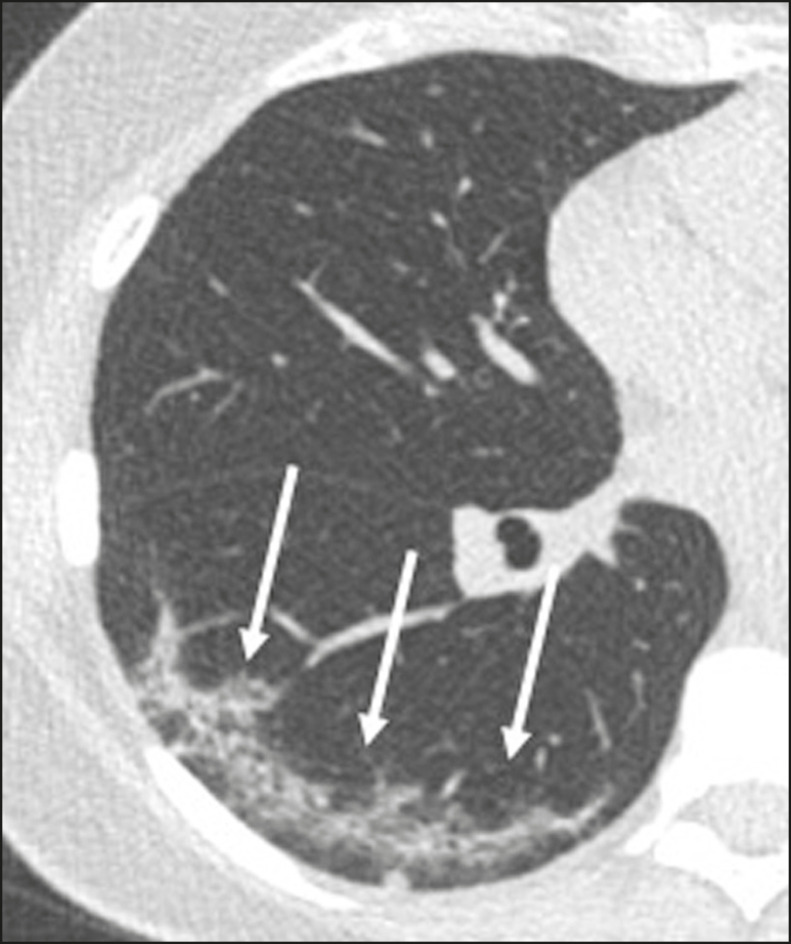
Chest CT scan showing a subpleural line (arrows) in a patient with a confirmed diagnosis of COVID-19 who presented with a 9-day history of dry cough, dyspnea, and anosmia.

**Figure 7:**
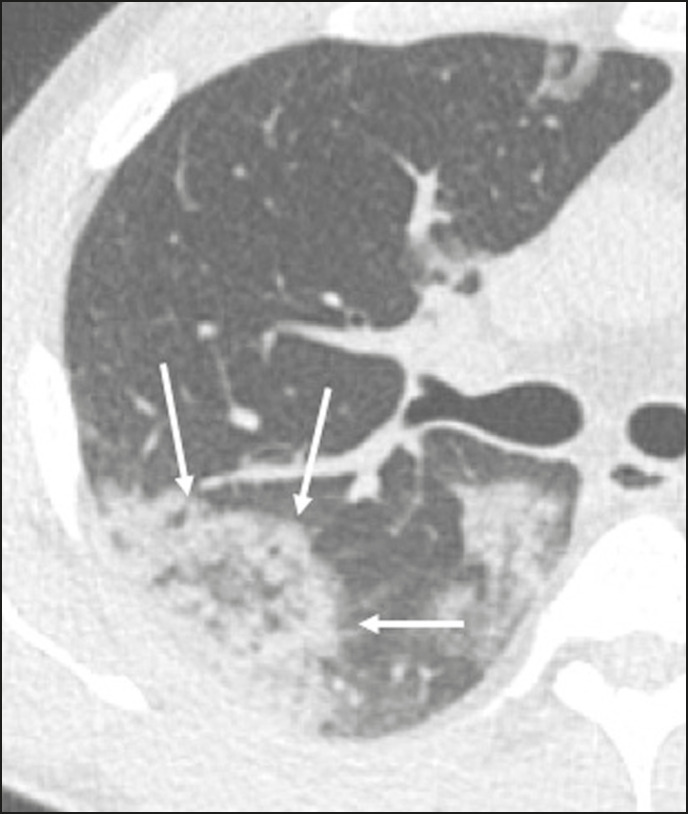
Chest CT scan of a 59-year-old male patient who was treated in the intensive care unit for 12 days with a diagnosis of COVID-19, showing pulmonary opacities, one with an inverted halo sign (arrows), characterized by consolidation with a low-attenuation center.

**Figure 8:**
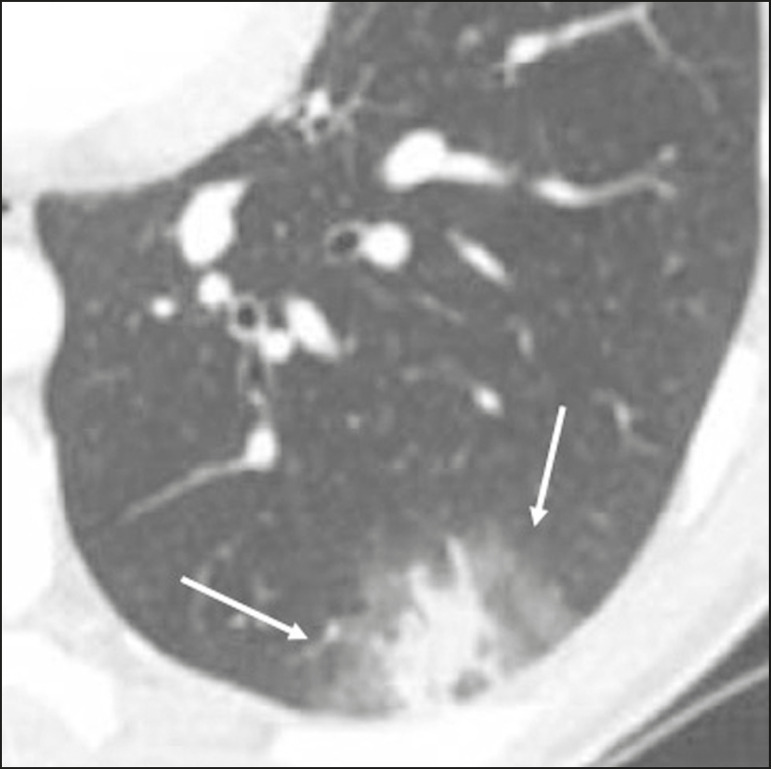
Chest CT scan showing a halo sign (arrows), characterized by pulmonary nodular consolidation with ground-glass halo (arrows), in a patient with COVID-19.

Ground-glass opacities (Figure 2) are found in 57-98% of patients with COVID-19 and constitute an early manifestation of the disease. They are generally bilateral, typically with a peripheral distribution in the lung bases, and have a rounded aspect in some cases^(19)^. A crazy-paving pattern (Figure 3) is seen in 5-89% of patients^(20,23)^, with a higher incidence during the peak phase of the disease (a period of approximately 10 days), and may be due to intralobular septal thickening caused by an inflammatory process induced by the infection^(23)^. Parenchymal consolidations (Figure 4) are present in 2-64% of patients, especially in those over 60 years of age, and indicate a more advanced phase of the disease, typically appearing 10-14 days after the onset of symptoms^(24)^. Reticular pulmonary opacities (Figure 5) are seen in 48% of patients, usually in more advanced phases of the disease and in individuals over 60 years of age. Subpleural lines (Figure 6), which can be indicative of pulmonary edema or progression to fibrosis, are observed in 20% of patients. They are more common in patients over 60 years of age and after 10 days of illness^(25,26)^. The inverted halo sign (Figure 7) is generally seen in later phases of the disease, occurs in approximately 4% of patients, and may be due to organizing pneumonia or pulmonary infarction^(24)^. Airway changes, such as air bronchograms, are uncommon and may indicate severity^(26)^. Bronchial thickening, bronchiectasis, and centrilobular nodules are rare in adults, being more common in pediatric cases of COVID-19^(27)^. The halo sign (Figure 8), defined as an area of ground-glass attenuation surrounding a focus of consolidation, is uncommon^(28)^, and the differential diagnosis should include other infections (mainly fungal infections), vasculitis, and neoplasms^(29)^.

**Figure 9:**
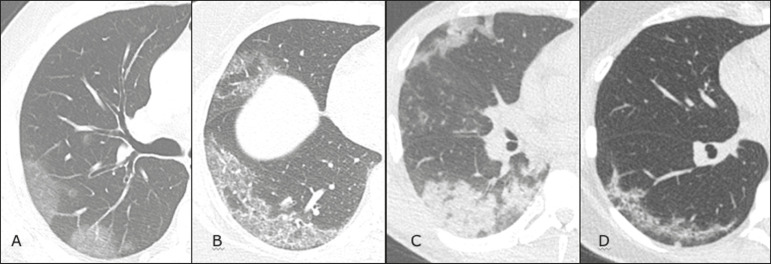
Sequential high-resolution CT images of patients with COVID-19, illustrating the different phases of the disease. A: Phase 1, with ground-glass pulmonary opacities. B: Phase 2, with mosaic pulmonary paving. C: Phase 3, with pulmonary consolidations. D: Phase 4, with reticular pattern.

Imaging findings on chest CT vary according to the evolutionary phase of the disease (Figure 9), categorized by the time since the onset of symptoms^(30)^:

- In the initial phase (0-4 days after symptom onset), ground-glass opacities can be seen, although the chest CT may be normal overall.
- In the progressive phase (5-8 days after symptom onset), chest CT shows diffuse ground-glass opacities, a crazy-paving pattern, and consolidation.
- In the peak phase (9-13 days after symptom onset), consolidation foci become more prevalent; diffuse ground-glass opacities and a crazy-paving pattern persist; and some residual parenchymal bands appear.
- In the absorption phase (≥ 14 days after symptom onset), there is gradual absorption of the consolidation foci, diffuse ground-glass opacities still being seen, and the crazy-paving pattern is no longer observed.

In addition to its applications for the diagnosis and monitoring of disease progression, CT can be used in the evaluation of complications, such as pulmonary thromboembolism, concomitant bacterial infection, lung abscess, pleural empyema, acute respiratory distress syndrome, myocarditis, and acute lung edema^(19,20)^.

### MRI of the chest

In relation to chest CT, MRI of the chest does not provide additional information for the assessment of pulmonary findings in patients with COVID-19, as well as being an imaging method that is less widely available, is more costly, and requires more time for image acquisition. The pulmonary alterations of COVID-19 on MRI follow the same distribution and aspect as those seen on X-rays and CT scans, MRI showing areas of high signal intensity in T1-weighted and T2-weighted sequences^(31)^. The principal indications for MRI in patients with COVID-19 are in the evaluation of complications such as myocarditis or acute necrotizing encephalopathy, both of which have been reported in some patients with the disease.

The pathogenesis of acute necrotizing encephalopathy in patients with COVID-19 has been linked to a cerebral cytokine storm, with disruption of the blood-brain barrier. On cranial CT, the imaging findings described are hypoattenuation of the medial portion of the thalamus, which shows a hyperintense signal in T2-weighted fluid-attenuated inversion recovery MRI sequences, as do the subinsular region and the medial portion of the temporal lobes, with gadolinium-enhancing ring lesions in gadolinium contrast-enhanced T1-weighted sequences^(32)^.

### PET/CT

Currently, the use of ^18^F-fluorodeoxyglucose PET/CT is not indicated for the evaluation of patients under clinical suspicion of COVID-19. Although the method produces the same findings as chest CT, it can also show functional changes, characterized by increased metabolic activity in the area of lung lesions, as well as in the lymph nodes of the pulmonary hila and mediastinum. Although ^18^F-fluorodeoxyglucose PET/CT may have future applications in the monitoring and prognostic evaluation of the disease, additional studies are needed in order to clarify such aspects^(33)^.

### Ultrasound of the chest

Although not part of the diagnostic algorithm of the Brazilian National Ministry of Health, point-of-care ultrasound (POCUS) can be used in some patients with suspected COVID-19. In a joint statement, the CBR and the Associação Brasileira de Medicina de Emergência (ABRAMEDE, Brazilian Association of Emergency Medicine) recommended the use of POCUS in certain situations^(34)^: when there is involvement of the lower respiratory tract (particularly in patients with extremely severe or unstable disease or in places where CT is unavailable); when there is acute clinical worsening (e.g., shock, respiratory failure, or both) in the emergency department, hospital wards, or intensive care units; when there is a need for central venous access, for which ultrasound-guided catheter placement increases the safety of the procedure and POCUS can be used in order to verify that it was successful. According to the CBR/ABRAMEDE statement^(34)^, the main POCUS findings are thickening of pleural lines, the presence of B lines, and peripheral pulmonary consolidations, A lines being seen in the recovery phase of the disease. The statement also recommends that, after each use, the ultrasound equipment be cleaned in accordance with the protocol of the local institution.

## QUANTIFICATION OF PULMONARY FINDINGS

Some articles have mentioned the role of X-ray and CT in quantifying the extent of pulmonary involvement or the aerated area of the lung parenchyma and have sought to correlate those findings with the clinical outcome. Currently, a large part of that evaluation is done in a visual, subjective way, resulting in great intraobserver and interobserver variability. At some centers^(35,36)^, parenchymal involvement has been quantified by the extent-as mild (< 25%), moderate (25-50%), or severe (> 50%)-or by the degree of lung aeration.

New tools have been developed for quantitative analysis of the impairment caused by pulmonary changes, in conjunction with artificial intelligence (AI) tools, and should be available for clinical use in the near future. According to the latest version of the CBR recommendations for the use of imaging methods in patients suspected of COVID-19^(11)^, the body of evidence in the literature does not support the visual/semi-quantitative or quantitative analysis by CT in patients with COVID-19 pneumonia.

## STRUCTURED REPORTS OF CHEST CT

A number of models for structured reporting of chest CT examinations have been proposed, chief among which is the COVID-19 Reporting and Data System (CO-RADS), proposed jointly by the Radiological Society of North America and the British Society of Thoracic Imaging^(37)^. The structured report proposed in the CO-RADS categorizes pulmonary findings into four patterns:

- Typical viral pneumonia pattern (Figure 10)-presence of bilateral, peripheral or multifocal, rounded ground-glass opacities (with or without consolidations and a crazy-paving pattern), together with the inverted halo sign or other signs of organizing pneumonia
  **Figure 10:**
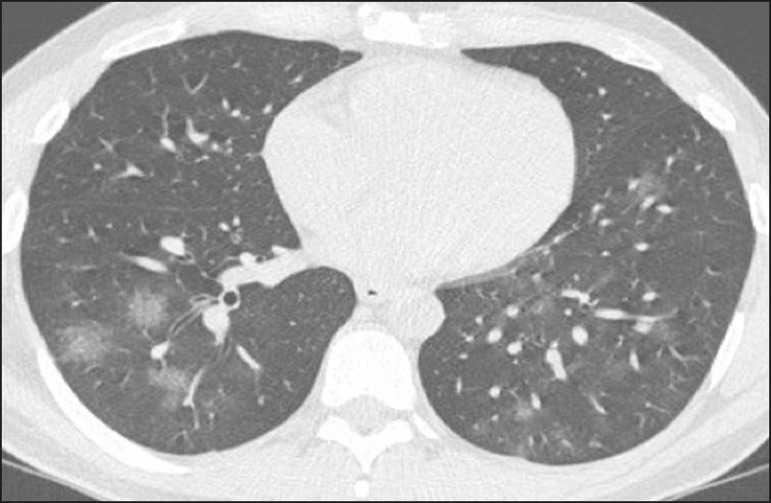
Typical viral pneumonia pattern for on high-resolution CT of the chest, with bilateral ground-glass opacities.
- Undetermined viral pneumonia pattern (Figure 11)-absence of typical findings and presence of ground-glass opacities with a unilateral, central, tenuous, diffuse, or atypical distribution
  **Figure 11:**
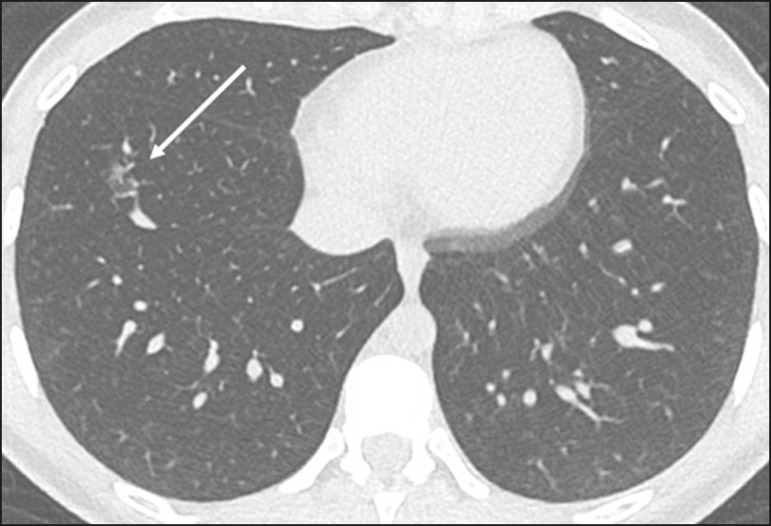
Undetermined viral pneumonia pattern on high-resolution CT of the chest, with tenuous unilateral ground-glass opacity (arrow).
- Atypical viral pneumonia pattern (Figure 12)-absence of typical or indeterminate findings and presence of lobar/segmental consolidation (without ground-glass opacities), septal thickening with pleural effusion, centrilobular micronodules, or cavities
  **Figure 12:**
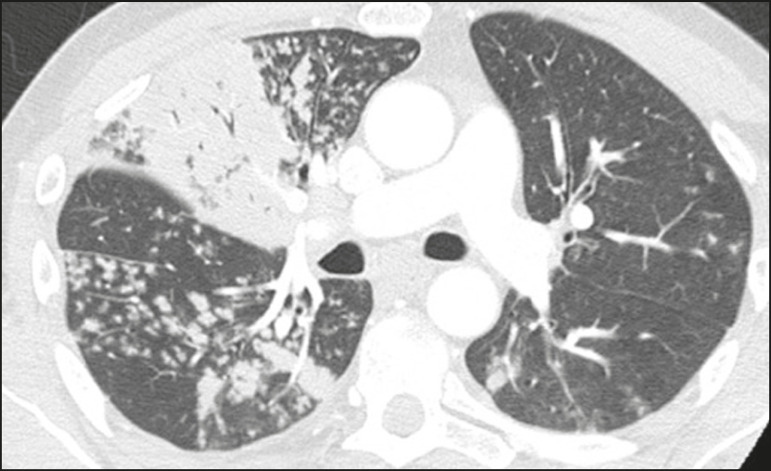
Atypical viral pneumonia pattern on high-resolution CT of the chest, with multiple centrilobular pulmonary nodules accompanied by parenchymal consolidation with air bronchograms. The patient had a confirmed diagnosis of pulmonary tuberculosis.
- Negative examination for viral pneumonia-examination without significant pulmonary changes or with changes unrelated to infection

The CO-RADS reporting model uses a numerical system to classify the changes found on chest CT. However, it still needs to be validated, because the original study had some limitations^(38)^, including the fact that it evaluated a small study group, representative of a population admitted to emergency departments and requiring hospital admission during the acute phase of the COVID-19 pandemic, as well as that it employed readers with limited experience in comparison with that of those in regions where the prevalence of the disease is high and that the diagnosis of the disease was based on the clinical impression alone in some cases, even when the RT-PCR result was negative.

## INNOVATIONS

Several innovations have emerged during the COVID-19 pandemic, not only for the diagnosis of the disease but also for the monitoring of infected individuals, patient care, and treatment^(39)^. For early detection of the disease, companies have sought solutions to track SARS-CoV-2-infected individuals by using devices such as watches and heart monitors or biosensors connected to cell phones, providing continuous data on electrocardiogram recordings, oxygen saturation, and analysis of breath. Some countries have monitored the population by using the Bluetooth technology employed in cell phones, notifying those who have had recent contact with individuals who later receive a confirmed diagnosis of COVID-19. Health startups also make it possible for individuals themselves, when they have suspected symptoms of COVID-19, such as coughing and shortness of breath, to upload recordings of their cough and breathing to a system that collects the information, analyzing it through AI and building predictive models.

New diagnostic tests have been developed in various countries, including Brazil, which has increased the processing capacity of the tests to detect SARS-CoV-2 through analyses with next-generation sequencing or using targeted proteomics based on mass spectrometry^(40)^. Other companies have invested in self-administered tests that individuals can take in their homes, with the objective of facilitating early diagnosis, not overburdening health care systems, reducing costs, and expanding the scale of testing overall.

There have been studies evaluating AI solutions based on X-ray or CT of the chest for the detection, diagnosis, and quantification of pulmonary findings related to viral infection, as well as for the prediction of complications, with the aim of promoting early treatment initiation. The greatest potential benefit of AI in COVID-19 may be the rapid screening of patients under clinical suspicion of having the disease, especially in places that have been severely affected by the pandemic, with many cases and few diagnostic resources^(41,42)^. However, AI-based solutions are still in the validation phase for examinations of patients suspected of having COVID-19, sometimes producing false results and not taking the place of evaluation by a radiologist, who performs an integrated analysis of clinical data, laboratory test results, and imaging findings. A virtuous scenario will be the union of the characteristics of these systems, such as detection and quantification of findings, with the best human qualities, such as intuition, common sense, and contextual analysis of the data.

Several national projects uniting multidisciplinary teams have been carried out in order to develop an automated tool capable of recognizing patterns of pulmonary changes on chest X-rays and chest CT scans of patients with COVID-19. The biggest challenge is the creation of a robust and reliable database of images and clinical data, analyzed and curated by radiologists with knowledge of annotation solutions for further analysis by AI.

During the COVID-19 pandemic, telemedicine has also been widely used to assist doctors and patients in maintaining care and for second opinions among medical teams, allowing individuals to receive health care and maintain the physical isolation necessary to contain the epidemic.

## CONCLUSION

The COVID-19 pandemic has altered the routine of human beings and the health care sector in a significant way. It is essential that radiologists be aware of the main clinical and imaging aspects of COVID-19, as well as guidelines for requesting and using imaging methods; measures for protecting patients and health professionals; systems for quantifying pulmonary findings and for the preparation of integrated reports; and the main innovations that have emerged during this pandemic.
